# Few chemoreceptor genes in the ambrosia beetle *Trypodendron lineatum* may reflect its specialized ecology

**DOI:** 10.1186/s12864-024-10678-4

**Published:** 2024-08-06

**Authors:** Twinkle Biswas, Heiko Vogel, Peter H. W. Biedermann, Maximilian Lehenberger, Jothi Kumar Yuvaraj, Martin N. Andersson

**Affiliations:** 1 Department of Biology, Lund University, Sölvegatan 37, 223 62 Lund, Sweden; 2 Department of Insect Symbiosis, Max Planck Institute for Chemical Ecology, Jena, Germany; 3 Chair of Forest Entomology and Protection, University of Freiburg, Stegen-Wittental, Germany; 4 Department of Biochemistry, Max Planck Institute for Chemical Ecology, Jena, Germany

**Keywords:** Coleoptera, Curculionidae, Scolytinae, Genome, Maximum-likelihood tree, Odorant receptor, Gustatory receptor, Ionotropic receptor, Mutualism, Fungus

## Abstract

**Background:**

Chemoreception is crucial for insect fitness, underlying for instance food-, host-, and mate finding. Chemicals in the environment are detected by receptors from three divergent gene families: odorant receptors (ORs), gustatory receptors (GRs), and ionotropic receptors (IRs). However, how the chemoreceptor gene families evolve in parallel with ecological specializations remains poorly understood, especially in the order Coleoptera. Hence, we sequenced the genome and annotated the chemoreceptor genes of the specialised ambrosia beetle *Trypodendron lineatum* (Coleoptera, Curculionidae, Scolytinae) and compared its chemoreceptor gene repertoires with those of other scolytines with different ecological adaptations, as well as a polyphagous cerambycid species.

**Results:**

We identified 67 ORs, 38 GRs, and 44 IRs in *T. lineatum* (‘Tlin’). Across gene families, *T. lineatum* has fewer chemoreceptors compared to related scolytines, the coffee berry borer *Hypothenemus hampei* and the mountain pine beetle *Dendroctonus ponderosae*, and clearly fewer receptors than the polyphagous cerambycid *Anoplophora glabripennis*. The comparatively low number of chemoreceptors is largely explained by the scarcity of large receptor lineage radiations, especially among the bitter taste GRs and the ‘divergent’ IRs, and the absence of alternatively spliced GR genes. Only one non-fructose sugar receptor was found, suggesting several sugar receptors have been lost. Also, we found no orthologue in the ‘GR215 clade’, which is widely conserved across Coleoptera. Two TlinORs are orthologous to ORs that are functionally conserved across curculionids, responding to 2-phenylethanol (2-PE) and green leaf volatiles (GLVs), respectively.

**Conclusions:**

*Trypodendron lineatum* reproduces inside the xylem of decaying conifers where it feeds on its obligate fungal mutualist *Phialophoropsis ferruginea.* Like previous studies, our results suggest that stenophagy correlates with small chemoreceptor numbers in wood-boring beetles; indeed, the few GRs may be due to its restricted fungal diet. The presence of TlinORs orthologous to those detecting 2-PE and GLVs in other species suggests these compounds are important for *T. lineatum*. Future functional studies should test this prediction, and chemoreceptor annotations should be conducted on additional ambrosia beetle species to investigate whether few chemoreceptors is a general trait in this specialized group of beetles.

**Supplementary Information:**

The online version contains supplementary material available at 10.1186/s12864-024-10678-4.

## Background

Deciphering chemical information in the environment is essential for the fitness of many animals. In insects, chemoreception (olfaction and taste/gustation) is often crucial for finding hosts, food, oviposition sites and mates, to maintain symbiotic relationships, and to avoid predators, competitors, non-hosts, and harmful microbes [1–4]. Chemoreception involves the binding of chemicals to membrane-bound receptor proteins that are encoded by some of the largest gene families in insect genomes [5–8]. The binding of a chemical to a receptor results in a conformational change that translates the chemical energy in the stimulus into a neuronal signal in the sensory neuron, which is conveyed to the central nervous system for interpretation [1].

The different families of insect chemoreceptors are the odorant receptors (ORs) [7], gustatory receptors (GRs) [9] and ionotropic receptors (IRs) [5, 10]. The ORs represent the primary means by which insects detect airborne chemicals [2]. These receptors are seven transmembrane domain proteins present in the dendrites of olfactory sensory neurons (OSNs) primarily in the antennae and maxillary palps. They form a heterotetrameric complex with a conserved odorant receptor co-receptor (Orco) [11–14], which is essential for the formation of an ion channel upon ligand-binding in the OR [15–17]. The more ancient family of GRs is believed to belong to the same superfamily as the ORs [18–20]. Gustatory receptors are expressed in gustatory as well as non-gustatory organs and are known to recognise non-volatile compounds including sugars, amino acids, bitter compounds, contact pheromones, as well as the gas carbon dioxide [6, 9, 21, 22]. Ionotropic receptors are related to ionotropic glutamate receptors (iGluRs) that have important functions in synaptic communication. Several of the conserved ‘antennal IRs’ are involved in olfaction, but others play a role in sensing humidity, salt and temperature [5, 23–26]. Members of the so-called ‘divergent IRs’ have been assigned a gustatory function [23, 27]. The IRs are three transmembrane domain proteins that function together with different IR co-receptors (IR8a, IR25a, and IR76b) [28, 29]. Unlike ORs, which detect a variety of compound structures, the olfactory IRs primarily detect short-chained compounds, such as organic acids and amines [29–32].

The divergent chemoreceptor gene families evolve according to a ‘birth and death’ model, in which gene duplication represents the birth of a gene, and pseudogenization or deletion the death [2, 33]. This mode of evolution has generated variation between species in the sizes of the chemoreceptor gene families, with different taxa displaying different extents of chemoreceptor lineage radiations and losses [33, 34]. However, our understanding of how the chemoreceptor gene families may expand or retract in relation to species ecology remains poorly understood. A previous study on wood-boring beetles from different taxonomic families proposed a positive correlation between host range and the number of chemoreceptor genes present in the genome. Specifically, fewer genes were annotated in the genomes of two stenophagous species (mountain pine beetle *Dendroctonus ponderosae* Hopkins, Curculionidae, and the emerald ash borer *Agrilus planipennis* Fairmaire, Buprestidae) than in a polyphagous species (Asian longhorn beetle *Anoplophora glabripennis* Motschulsky, Cerambycidae) [35]. However, the generality of this hypothesis was recently challenged by the finding that the genome of the highly specialized Western corn rootworm (*Diabrotica virgifera virgifera* LeConte, Chrysomelidae) contains expanded families of both ORs and IRs compared to several beetle species with broader host ranges [36].

To understand how the evolution of the chemoreceptor gene families may relate to ecological specializations, these genes should be manually annotated from genomes because annotations of tissue-specific transcriptomes, which is more common [37], as well as automated genome annotation pipelines, typically miss a significant fraction of the chemoreceptors encoded by the genome [34, 35, 38–40]. Most such genome-scale efforts have targeted various species of Diptera, Hymenoptera and Lepidoptera, [18, 41–49], whereas less focus has been on the Coleoptera, the largest order of insects with > 400,000 described species. Among the currently existing studies, the OR family has received most attention [34], whereas the GR and/or IR families have to our knowledge only been completely annotated in seven species: the red flour beetle *Tribolium castaneum* Herbst (Tenebrionidae) [50, 51], *A. glabripennis* [52], the Colorado potato beetle *Leptinotarsa decemlineata* Say (Chrysomelidae) [39], *D. ponderosae*, *A. planipennis* [35], *D. v. virgifera* [36], and the coffee berry borer *Hypothenemus hampei* Ferrari (Curculionidae) [53] (see also Table 1). Hence, annotation of the chemoreceptor genes in additional species is required to improve our understanding of the molecular evolution of chemoreception in this diverse insect order. To this end, targeting the chemoreceptor genes from related species that differ in ecological traits may be particularly rewarding, because the species phylogeny is known to at least partly dictate the genomic chemoreceptor gene content, including the presence and phylogenetic distributions of receptor lineage radiations and losses [34, 54].

**Table 1:** The number of chemoreceptor genes (including pseudogenes) manually annotated from genomes of coleopteran species (see main text for references)

Species	Odorant receptors	Gustatory receptors	Ionotropic receptors	Total
*Trypodendron lineatum*^a^	67	38	44	149
*Dendroctonus ponderosae*	86	60	57	203
*Hypothenemus hampei*	67	66	33	166
*Anoplophora glabripennis*	132	234	72	438
*Leptinotarsa decemlineata*	80	147	27	254
*Tribolium castaneum*	338	245	80	663
*Agrilus planipennis*	47	30	31	108
*Diabrotica v. virgifera*	160	N/A	107	N/A

^a^This study

In this study, we sequenced the genome of the striped ambrosia beetle, *Trypodendron lineatum* Olivier (Coleoptera, Curculionidae, Scolytinae), and manually annotated the three chemoreceptor gene families from the resulting assembly for comparison with related, and less related, species with different ecological adaptations. Ambrosia beetles are a polyphyletic group with more than 3,400 species from at least 11 lineages within the Platypodinae and Scolytinae subfamilies that have evolved obligate nutritional mutualism with filamentous fungi on several independent occasions [55–58]. To the best of our knowledge, the chemoreceptor genes have so far not been annotated from a genome of any ambrosia beetle species. *Trypodendron lineatum* is a Holarctic wood-boring pest that attacks dead or dying conifer trees, decreasing wood quality, and thus causing economic damages [59–61]. It belongs to the same subfamily as *D. ponderosae* and *H. hampei*, which both have their chemoreceptor genes annotated from genomes. *Trypodendron lineatum* is attracted to volatiles from dead or decaying conifer trees [62–65], and avoids non-host volatiles from angiosperm plants [66, 67]. Upon finding a suitable host, *T. lineatum* females release the aggregation pheromone ( +)-lineatin (3,3,7-trimethyl-2,9-dioxatricyclononane) to attract conspecifics [68]. Afterwards, they construct a tunnel system in the xylem, in which the walls are inoculated with a community of fungi dominated by the nutritional fungal mutualist *Phialophoropsis ferruginea* (Mathiesen-Käärik) (Ascomycota) serving as the primary food source for the developing beetle offspring [69–71]. The ecologies of the two scolytines *D. ponderosae* and *H. hampei* are different from the rather secondary fungus-farming lifestyle of *T. lineatum*. *Dendroctonus ponderosae* is an aggressive bark beetle with either an obligate or a facultative mutualism with varying fungal species depending on geographic range (such as *Grosmannia clavigera* (Rob.-Jeffr. & Davidson) Zipfel, Z.W. de Beer & M.J. Wingf., *Ophiostoma monitum* (Rumbold) von Arx, *Leptographium longiclavatum* Lee, Kim & Breuil (all Ascomycota), and *Entomocorticium dendroctoni* Whitn., Band. & Oberw. (Basidiomycota)) [72–75]. The beetles bore into and feed on the phloem (not the xylem) and supplement their diet with the fungi; together the beetles and the fungi are able to kill healthy pine trees of several species in North America [76–78]. On the other hand, *H. hampei* is a spermatophagous beetle that spends most of its life cycle inside the seeds of coffee berries and has no known mutualism with fungi [79, 80]. In addition to these two scolytine species, we further included the chemoreceptors of the more distantly related and polyphagous wood-boring cerambycid *A. glabripennis*, which is able to attack many different species of plants [81, 82] to provide an expanded comparative overview. Due to the specialized ecology and narrow diet of *T. lineatum*, we hypothesized that it may have comparatively few chemoreceptor genes in its genome.

## Results

### Genome assembly

We used Nanopore technology to sequence the genome of male (the heterogametic sex) *T. lineatum*. The total genome assembly size was 83.6 Mbp, divided between 832 contigs with a contig N_50_ size of 915 kbp. The longest contig was 4.16 Mbp. The BUSCO analysis indicated a genome completeness of 97.9% (1338 of 1367 BUSCOs were present as complete genes), with 96.3% of the genes being present as complete single copy orthologues, 1.6% as complete duplicated orthologues, and 1.2% were fragmented. Only 0.9% of the BUSCOs (13 genes) were considered entirely missing from the assembly. Overall, these results suggest a rather small but complete genome assembly, suitable for annotation of the chemoreceptor gene families. A total of 149 chemoreceptor genes from three gene families were annotated in the present genome assembly (Table 1; amino acid sequences, contig positions, and additional annotation details are provided in Additional File 1). We also sequenced and assembled an antennal transcriptome (see Methods for details), which was used to support the genome annotations and to investigate whether the genes are expressed in the main chemosensory organ. Of the 149 annotated chemoreceptor genes, 130 (87%) were recovered as full-length or partial transcripts in this assembly (Additional File 1).

### Odorant receptors (ORs)

A total of 67 TlinORs, including Orco, were annotated in the *T. lineatum* genome (Table 1). Four of these genes were regarded as pseudogenes, and another four gene models remain partial because the N-terminal exon(s), which is typically divergent, could not be confidently identified. Six OR genes contained sequencing-induced indels, which were manually corrected using raw Illumina reads (see Methods). Apart from TlinOR55NTE and TlinOR32PSE (gene name suffixes are explained in the Methods), which comprised 306 and 303 amino acids, respectively, all ORs exceeded 370 amino acids, including the partial genes and pseudogenes. Some of the OR genes were found in tandem arrays in the assembly, with the largest ones each presenting three genes close together (TlinOR14-16, TlinOR57-59, TlinOR63-65). Among the full-length OR genes, the number of introns varies from three to seven (typically ranging from 50 to 150 bp in size), except for the Orco gene that contains 10 introns. Fifty-eight (87%) of the 67 OR genes that were annotated in the genome could be confirmed as full-length or partial transcripts in the antennal transcriptome assembly (Additional File 1).

**Fig. 1:**
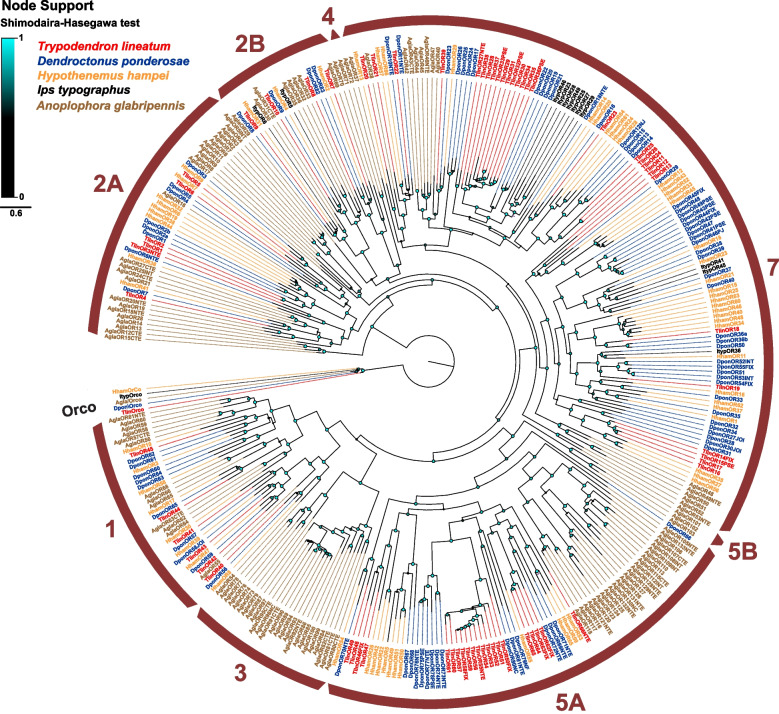
Approximately maximum likelihood phylogenetic tree of odorant receptors (ORs). Included are OR amino acid sequences from the genomes of *Trypodendron lineatum* (Tlin; red), *Dendroctonus ponderosae* (Dpon; blue), *Hypothenemus hampei* (Hham; orange), and *Anoplophora glabripennis* (Agla; brown), as well as functionally characterized ORs from *Ips typographus* (Ityp; black). The tree is rooted with the conserved lineage of Orco proteins and is based on a MAFFT alignment. The coloured nodes (cyan to black) indicate SH (Shimodaira-Hasegawa) local branch support, which increases with the size and brightness of the circles. The arcs indicate the different major groups of coleopteran OR families [34]. The Methods section provides a full description of receptor suffixes and the sources of sequence data

A previous study that phylogenetically analysed the ORs from ten beetle genomes defined nine major monophyletic OR subfamilies in Coleoptera, and these subfamilies were named Group 1, 2A, 2B, 3, 4, 5A, 5B, 6, and 7 [34]. Several of these subfamilies had been recognized also previously [40, 50]. In the present study, the phylogenetic OR analysis included the receptors in the genomes of the curculionids *T. lineatum*, *D. ponderosae*, and *H. hampei*, and the cerambycid *A. glabripennis*, as well as the functionally characterized ORs from the Eurasian spruce bark beetle *Ips typographus* L. (Curculionidae, Scolytinae) (Fig. 1). This analysis showed that, similar to other curculionids, most of the ORs in *T. lineatum* belong to Group 7 (29 ORs), followed by Group 5A (21 ORs), Group 1 (6 ORs), Group 2B (6 ORs) and 2A (4 ORs). Furthermore, like other curculionids, *T. lineatum* entirely lacks ORs from Groups 3, 4, 5B and 6. It is also evident from our analysis that the phylogenetic distribution of ORs in *T. lineatum* (and the other curculionids) is different from that in *A. glabripennis*, which has a large proportion of ORs in Groups 2A, 2B and 3, representatives also in Groups 4 and 5B, and comparatively few ORs in Group 7. In *T. lineatum*, the largest species-specific OR radiation contains 12 ORs in Group 5A (TlinOR50-61) and the second largest comprise 11 ORs in Group 7 (TlinOR28-38). Nineteen cases of supported (Shimodaira-Hasegawa [SH] support value > 0.7) simple (1:1) orthologous relationships between TlinORs and ORs in one or more of the bark beetle species were identified (Additional File 2), including two TlinORs grouping with functionally characterized ORs in *D. ponderosae* and *I. typographus* within Group 2A: TlinOR9 (and HhamOR31) grouping with ItypOR6 and DponOR8, both responding to 2-phenylethanol, and TlinOR10 (and HhamOR4) grouping with ItypOR5 and DponOR9, both responding to C6 green leaf volatiles (GLVs) (Fig. 1) [83]. No clear orthologous relationships were found between ORs in any of the curculionid species and *A. glabripennis.* Similar to a previous study that also analysed ORs from only curculionid and cerambycid species [84], our tree did not separate OR groups 2A and 2B as monophyletic, which is likely due to the narrow taxonomic range included in the analysis.

### Gustatory receptors (GRs)

We annotated 38 GR genes in *T. lineatum*, including five putative fragmented pseudogenes with e.g. missing N- or C-termini, missing internal sequence, frameshifts, and/or several internal stop codons (Additional File 1). The total number of TlinGRs is lower as compared to the two other scolytines in the comparison (Table 1). Among the 33 putatively functional GR genes, all but one could be annotated to full length, with the C-terminal exon missing from TlinGR32CTE. Six of the putatively functional GR genes contained sequencing-induced indels in exons which could be corrected with support from the raw Illumina reads. Whereas the GRs putatively detecting carbon dioxide (GR1-3) or sugars, including the fructose receptor (GR4-5), contain four to seven introns (typically 50–150 bp), most of the putative bitter taste GRs have only one intron (Additional File 1). However, nine of them have additional introns with the largest number (six) found in TlinGR6. Alternative splicing is common among insect GR genes, including in related scolytine beetles [35, 53]. Most commonly, the alternatively spliced GR genes previously observed in *D. ponderosae* have two exons, with the different splice variants sharing the second exon but alternating the first one. This has been evident from the gene structure by the presence of two or several alternative first exons (e.g., exons 1A and 1B, and sometimes also 1C, and 1D) located in tandem array, followed by a single C-terminal exon (exon 2) [35]. None of the annotated TlinGR genes showed such a structure, suggesting that the annotated genes are not alternatively spliced*.* A few of the TlinGR genes were found in tandem arrays on contigs with the largest array presenting three genes (TlinGR8-10). Thirty-one (82%) of the 38 GR genes that were annotated in the genome could be confirmed as full-length or partial transcripts in the antennal transcriptome assembly (Additional File 1).

**Fig. 2:**
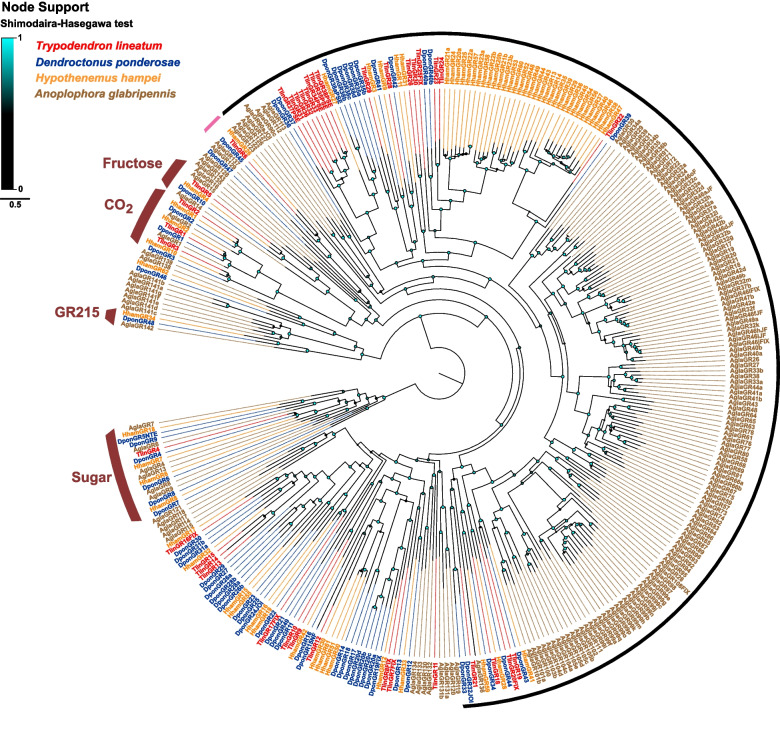
Approximately maximum likelihood phylogenetic tree of gustatory receptors (GRs). Included are GR amino acid sequences from the genomes of *Trypodendron lineatum* (Tlin; red), *Dendroctonus ponderosae* (Dpon; blue), *Hypothenemus hampei* (Hham; orange), and *Anoplophora glabripennis* (Agla; brown). The tree is rooted with the ancestral lineage of sugar receptors and is based on a MAFFT alignment. The coloured nodes (cyan to black) indicate SH (Shimodaira-Hasegawa) local branch support, which increases with the size and brightness of the circles. The well-established GR clades with strong support across all four beetle species are represented by thick red arcs. The thin black arc indicates a highly supported clade with differences between species in the extent of GR-lineage radiations. The pink arc indicates a clade with simple orthologues in both the curculionid and cerambycid species. The Methods section provides a full description of receptor suffixes and the sources of sequence data

The GRs from *T. lineatum* were phylogenetically analysed together with the GRs from *D. ponderosae*, *H. hampei* and *A. glabripennis*. This analysis showed that the three CO_2_ receptors (TlinGR1-3) are conserved in *T. lineatum*, as expected by their presence in other coleopterans (Fig. 2). In contrast, only one receptor (TlinGR4) is present within the non-fructose sugar receptor clade, and it is orthologous to DponGR4, HhamGR7, and AglaGR4. Because these other species each have several sugar receptors (6 DponGRs, 4 HhamGRs, and 6 AlgaGRs), this result suggests that *T. lineatum* has lost several sugar receptor genes. As for the other two curculionids, *T. lineatum* has one receptor (TlinGR5), orthologous to DponGR10 and HhamGR4, within the putative fructose receptor clade (*A. glabripennis* has three GRs in this clade). A previous study found one small clade among the putative bitter taste GRs that appear widely conserved across different families of Coleoptera, with a single orthologue in e.g., *T. castaneum*, *A. planipennis*, *A. glabripennis*, and *D. ponderosae* [35]. This clade was previously named the ‘GR215’ clade after the orthologue in *T*. *castaneum* (TcasGR215). Surprisingly, we did not find any *T. lineatum* GR that groups in this clade, suggesting it has been lost in this species. The putative bitter taste gustatory receptors (GRs) demonstrated species-specific expansions in each of the four species included in the analysis, varying from small to large clades. However, no exceedingly large expansion was evident in *T. lineatum*; the largest TlinGR-specific clade contained eight GRs, of which four are pseudogenes (Fig. 2). This contrasts with *H. hampei*, and particularly *A. glabripennis*, which both have much larger GR-lineage expansions. Among the putative bitter taste GRs, nine of the TlinGRs have supported simple orthologues in one or both other curculionids, whereas one orthologue is shared between *H. hampei*, *D. ponderosae*, *T. lineatum* and *A. glabripennis* (Fig. 2, Additional File 2)*.*

### Ionotropic receptors (IRs)

**Fig. 3:**
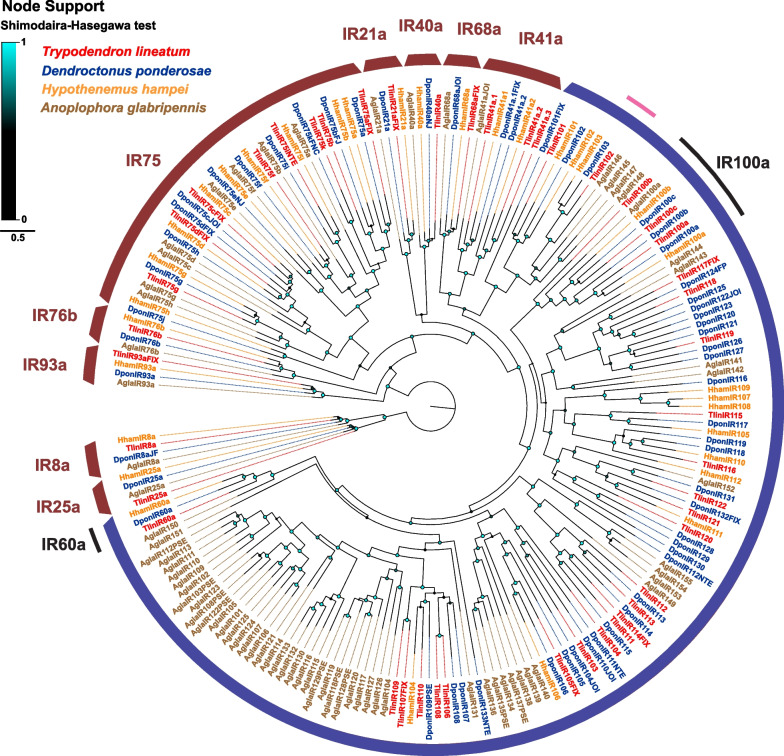
Approximately maximum likelihood phylogenetic tree of ionotropic receptors (IRs). Included are IR amino acid sequences from the genomes of *Trypodendron lineatum* (Tlin; red), *Dendroctonus ponderosae* (Dpon; blue), *Hypothenemus hampei* (Hham; orange), and *Anoplophora glabripennis* (Agla; brown). The tree is rooted with the conserved lineages of IR8a and IR25a proteins and is based on a MAFFT alignment. The coloured nodes (cyan to black) indicate SH (Shimodaira-Hasegawa) local branch support, which increases with the size and brightness of the circles. The well-established and widely conserved antennal IR clades are indicated by thick red arcs. The thick blue arc indicates the divergent IRs. The thin black arcs indicate highly supported clades of IR60a and IR100a that are conserved among the divergent IRs, and the thin pink arc indicates a newly observed clade with simple orthologous in both the curculionid and cerambycid beetles. The Methods section provides a full description of receptor suffixes and the sources of sequence data

A total of 44 TlinIR genes were annotated (Table 1), including 43 full-length genes and one partial gene (TlinIR75iNTE), for which the two N-terminal exons could not be found (Additional File 1). No pseudogenes were discovered among the IRs. As expected, members of all conversed antennal IRs (IR8a, IR21a, IR25a, IR40a, IR41a, IR68a, IR76b, IR93a, and IR75 members) were present in *T. lineatum*. For IR41a, the *T. lineatum* genome presented three paralogues close to each other on the same contig (separated by 455 bp and 242 bp, respectively) compared to two paralogues in both *D. ponderosae* and *H. hampei*, and a single gene in *A. glabripennis* (Fig. 3). Eight members of the IR75 family were identified compared to eleven in *D. ponderosae*, nine in *H. hampei*, and eight in *A. glabripennis*. All TlinIR75 members have corresponding orthologues in either *D. ponderosae* or *H. hampei* and, in most cases, both species. Among the divergent group of IRs, a single orthologue of IR60a and three paralogues in the IR100a clade were identified, like in *D. ponderosae.* No major radiation of divergent IRs was evident in the study species, which contrasts with the large radiations seen especially in *A. glabripennis.* Among the divergent IRs (apart from IR60a and the IR100a members), 12 examples of simple 1:1 orthologous relationships were evident between *T. lineatum* and *D. ponderosae* and/or *H. hampei* (Additional File 2). In fact, one example of simple orthology extending also to the Cerambycidae was evident, with very high support (SH = 1.0) for the close grouping of TlinIR102, DponIR103, HhamIR103, and AglaIR146 (Fig. 3, Additional File 2). In case of conserved antennal IRs, most of the TlinIRs have several introns with TlinIR40a having the largest number (15), whereas the divergent IRs are either intronless (15 IRs) or contain 1–3 introns (10 IRs) (Additional File 1). Previous genomic annotations of beetle IRs have unravelled introns in several of the conserved antennal IRs spanning several kilobases of sequence; for example, the two largest introns in DponIR93a cover > 18 kbp and > 15 kbp, respectively, with the whole gene from start to stop codon covering approx. 60 kbp of the genome [35]. In contrast, the largest of the 14 introns in TlinIR93a is only 155 bp, and with most introns ranging between approx. 50 to 70 bp. This is the case also for the other antennal IRs in *T. lineatum*, except for one 3 kbp intron in TlinIR8a, and one 1 kbp intron in TlinIR60a. In fact, the occurrence of large introns in *T. lineatum* was very rare across all annotated chemoreceptor gene families. Forty-one (93%) of the 44 IR genes that were annotated in the genome could be confirmed as full-length or partial transcripts in the antennal transcriptome assembly (Additional File 1).

## Discussion

We annotated 149 chemoreceptor genes from the genome of the ambrosia beetle *T. lineatum*, including 67 ORs, 38 GRs, and 44 IRs. A total of 138 of these genes could be annotated to full length, and 130 of them were recovered as full-length or partial transcripts in an antennal transcriptome; hence, the chemoreceptor gene set reported in this study is of high quality, facilitating future functional studies. The chemoreceptor genes had previously been manually annotated from the genomes of two species in the Scolytinae subfamily, *D. ponderosae* [35] and *H. hampei* [53], but to our knowledge, never from an ambrosia beetle. Hence, this study allows for improved analysis of chemoreceptor gene evolution within the Scolytinae subfamily, including comparisons between related species with different ecological specializations.

The total number of chemoreceptors in *T. lineatum* is clearly lower than in *D. ponderosae* (203 receptors), and slightly lower than in *H. hampei* (166 receptors). Compared to the polyphagous cerambycid wood-borer *A. glabripennis* (438 receptors) all three stenophagous scolytines have vastly fewer chemoreceptors (Table 1). A previous study showed that the wood-boring specialist *A. planipennis* also has a reduced chemoreceptor repertoire (108 receptors) compared to polyphagous species (Table 1). This suggests that a narrow host range/diet may correlate with low numbers of chemoreceptors in wood-boring beetles [35]. However, this trend may not be generally applicable when comparing with non-wood-boring beetle species [36]. The comparatively low number of chemoreceptors in *T. lineatum* is largely explained by the scarcity of large receptor lineage radiations, especially among the putative bitter taste GRs and the divergent class of IRs. This finding is reflected in the lack of larger tandem arrays of such chemoreceptors in the genome, with the largest ones encompassing only three genes assembled nearby on the same contigs. The largest TlinGR-radiation contained (only) eight receptors, of which four were fragmented pseudogenes. Additionally, none of the annotated TlinGR genes showed evidence of alternative splicing, which contributes to the comparatively low number of chemoreceptor proteins observed in this species. Alternative GR gene splicing has been found in both *D. ponderosae* and *H. hampei* [35, 53], and is highly prevalent in *A. glabripennis* [52]. Among the analysed scolytine species, *T. lineatum* has fewer chemoreceptor genes than *D. ponderosae* in all three gene families; however, compared to *H. hampei*, the ambrosia beetle presents the same number of ORs and a larger number of IRs (Table 1). However, the GR repertoire in *T. lineatum* is clearly smaller with only 38 receptors identified, compared to 60 and 66 GRs in the other two scolytine species, respectively. Surprisingly, the total assembly size of the *T. lineatum* genome at 83.6 Mbp is much smaller than the estimated genome sizes of other sequenced scolytine species, ranging from 163 Mbp in *H. hampei* to 373 Mbp in *Dendroctonus valens* [53, 85–87]. Nevertheless, our BUSCO analysis showing 97.9% completeness speaks in favor of a rather complete assembly and suggests that the comparatively few chemoreceptor genes identified is unlikely to be caused by parts of the genome being missing from the assembly. Also, the rare occurrence of chemoreceptor gene introns larger than 150 bp in *T. lineatum* is in stark contrast to e.g., *D. ponderosae* in which several of the genes have introns spanning several kilobases of sequence [35]. This compact gene structure is likely to contribute to the small assembly size of the *T. lineatum* genome.

We speculate that the observed differences in chemosensory gene content between the three scolytine species may relate to differences in their chemical ecologies, including associations with symbiotic fungi and complexity of pheromone communication systems. Most species of bark- and ambrosia beetles have symbiotic associations with microbes where especially the filamentous fungi are beneficial for the beetle host in terms of detoxifying conifer tree chemical defenses or by serving as an important food source [4, 88–92]. The different species of fungi emit different volatile blends, which are often attractive to the beetles, suggesting that chemoreception is important for the maintenance of the beetle-microbe association [4, 92–94]. Indeed, both *I. typographus* and *T. lineatum* have several OSN types (hence ORs) that are tuned to volatiles emitted by their respective fungal symbionts [4, 93]. Bark beetles, including *D. ponderosae* and *I*. *typographus*, both feed on a mixture of fungi and host tree phloem containing toxic compounds (e.g., terpenoids and polyphenols) [4, 72, 74, 77, 95–97], whereas the fungal mutualist *P. ferruginea* is likely the primary food source of *T. lineatum* [69–71]. We hypothesize that this specific diet may relate to the comparatively few bitter taste GRs in the ambrosia beetle. For comparison, polyphagous moth species have much larger numbers of bitter taste GRs as compared to monophagous or oligophagous species [98–100], and polyphagy has also been suggested to be linked to the very large GR count (522 GRs) in the omnivorous American cockroach *Periplaneta americana* L. [101]. Although speculative, it is possible that the loss of several sugar receptors in *T. lineatum* also is related to its restricted fungal diet. Losses of sugar receptors have previously been found in the herbivorous Hessian fly, *Mayetiola destructor* Say (Diptera; Cecidomyidae), which specializes on wheat and a few related grasses [46], and in some species of subterranean beetles within the tribe Leptodirini (Coleoptera; Leiodidae) [102]. Also, the lack of receptor lineage radiations among the divergent IRs in *T. lineatum*, which are putative taste receptors [23, 27], might relate to its specific diet. In contrast to *T. lineatum*, the coffee berry borer *H. hampei* has a large expansion of putative bitter taste GRs and several more sugar receptors. This difference may relate to its unique lifecycle inside coffee berries in which the developing coffee beans (containing several bitter compounds, [103]) are attacked and destroyed [79, 104, 105]. In terms of pheromone communication, bark beetles, including *D. ponderosae* and *I. typographus* are known to detect and respond to multiple compounds produced by both con- and heterospecific beetles [106–111]. In contrast, *T. lineatum* has a single component aggregation pheromone and electrophysiological studies have revealed that the majority of the common scolytine pheromone compounds are not detected by this species [93, 112], which may contribute to the fewer ORs compared to both *D. ponderosae* and *I. typographus* (73 ORs identified from an antennal transcriptome of this species; [84]). Similarly, it remains unknown whether pheromone communication exists in *H. hampei* [79, 113, 114], and if absent, it may also partly explain why this species has fewer ORs than the above-mentioned conifer feeding bark beetles.

The phylogenetic distribution of the *T. lineatum* ORs among the nine major monophyletic coleopteran OR Groups [34] was similar to other curculionids with ORs present in Groups 1, 2A, 2B, 5A and 7 (Fig. 1), and with the two latter groups containing the largest expansions. Several ORs from Group 7 have previously been functionally characterised from the Curculionidae family, including eight ORs from *I. typographus* [84, 115, 116], three ORs from the red palm weevil *Rhynchophorus ferrugineus* Olivier [117–119], two ORs from each of *D. ponderosae* and the pine weevil *Hylobius abietis* L. [83], and one OR from the rice water weevil *Lissorhoptrus oryzophilus* Kuschel [120]. We found 19 groups of simple 1:1 OR orthologues that were shared amongst the analysed scolytines, which is similar to observed results from studies targeting other species in this subfamily [53, 84]. Two of the TlinORs (TlinOR9 and TlinOR10) are orthologous to ORs that respond to 2-phenylethanol and GLV alcohols, respectively, in each of *I. typographus*, *D. ponderosae*, and *H. abietis* [83]*.* Due to the highly conserved responses in these functionally characterized receptors, it is possible that the ORs detect the same compounds in *T. lineatum.* Indeed, OSNs responding to GLVs and 2-phenylethenol have previously been identified in *T. lineatum*, although the responses to the latter compound were comparatively weak [93, 112]*.* Both *I. typographus* and *T. lineatum* have OSNs that specifically detect lanierone [93, 116], a pheromone compound used by some North American bark beetle species in the *Ips* genus [121]. In *I. typographus*, lanierone is detected by ItypOR36, and the compound reduces the attraction to traps baited with the aggregation pheromone in the field [116]. Surprisingly, no *T. lineatum* OR was orthologous to ItypOR36, which suggests that the OR that detects lanierone in *T. lineatum* probably has a different evolutionary origin. In contrast to ORs in Group 7, no OR from Group 5A has been functionally characterised in any beetle species, and previous studies have shown that the OR genes within this group are poorly expressed in the antennae; hence their potential roles in beetle olfaction remain unclear [34, 50, 84].

As with the ORs, several simple orthologous relationships were found among the scolytine putative bitter taste GRs and divergent IRs. In contrast to the ORs, however, in each of these gene families we observed one case where the orthology was extended to also include a receptor from the cerambycid *A. glabripennis* (TlinGR6, HhamGR26, DponGR45, AlgaGR137, Fig. 2; TlinIR102, HhamIR103, DponIR103, AglaIR142, Fig. 3; Additional File 2). Surprisingly, none of the TlinGRs were associated with the broadly conserved GR215 clade, which has members from several coleopteran families, including other scolytine species [35], indicating yet another GR loss in *T. lineatum.* Among the antennal IRs, we found a comparatively high number (8) of TlinIR75 members, which is common in scolytine beetles, and all have orthologues in the other scolytine species. Whereas IR75 receptors detect acids in in dipterans and lepidopterans [30, 31, 42, 122, 123], nothing is known about their functions in Coleoptera. Future research should aim to functionally characterise the chemoreceptors that are conserved in beetles to understand their role in beetle chemical ecology.

## Conclusions

We sequenced the genome of *T. lineatum* and subsequently manually annotated the OR, GR and IR genes from the assembly, representing the first genomic annotation of chemoreceptors in ambrosia beetles. We found that *T. lineatum* overall has fewer chemoreceptors than two other similarly analysed scolytine species, which is mainly attributed to a scarcity of large receptor-lineage radiations, especially noticeable among the putative bitter taste GRs and divergent IRs, and the absence of alternative splicing of chemoreceptor genes. Whereas the three carbon dioxide receptors were present, *T. lineatum* appears to have lost all but one (non-fructose) sugar receptor, and it lacks a member in the GR215 clade, which is broadly conserved across Coleoptera. We speculate that the comparatively small complement of chemoreceptors, particularly GRs, relates to its specialized ecology and intimate relationship with a single known fungal nutritional mutualist. We also found that *T. lineatum* has two ORs that are orthologous to ORs in other curculionids, responding to 2-phenylethanol and GLVs, respectively. Future studies should aim to functionally characterize these ORs also in *T. lineatum* to investigate if their functions are conserved as well in this species. Overall, this study lays an important foundation for future functional characterization of chemoreceptors in ambrosia beetles, which is important for advancing our understanding of the chemical ecology of these insects and potentially for facilitating future control of this damaging forest pest. Finally, our annotations revealed a compact chemoreceptor gene structure, dominated by short introns. This finding is strikingly different from other analysed coleopterans, including other scolytines. The potential functional consequences of this organizational difference remain unknown and raise general questions about insect genome and multi-gene family evolution, which should be addressed in future studies.

## Methods

### Genome sequencing and assembly

Genomic DNA (gDNA) was extracted from five male *T. lineatum* beetle specimens (collected using pheromone traps in a Norway spruce, *Picea abies* (L.) H. Karst, dominated forest in Tågaröd, South Sweden) using the Nanobind Big DNA kit (Circulomics, Baltimore, MD, USA) followed by enrichment for HMW (high molecular weight) DNA using the Short Read Eliminator kit XS (Circulomics), following the manufacturer’s instructions. Isolated HMW DNA purity and concentrations were measured using Nanodrop (Thermo Fisher, Waltham, MA, USA) and Qubit (Thermo Fisher). End-DNA repair was performed before library preparation using the NEBNext Ultra II DNA Library Prep Kit (New England Biolabs, Ipswich, MA, USA), following the manufacturer’s instructions. Sequencing adapters were then ligated using the Ligation Sequencing Kit (Oxford Nanopore Technologies, Oxford, UK), followed by a clean-up step with AMPure XP beads (Beckman Coulter). Sequencing was performed on a MinION platform (Oxford Nanopore Technologies). Priming of the MinION flow cells (vR9.4.1) for sequencing was performed with the Flow Cell Priming Kit (Oxford Nanopore Technologies), and flow cells were loaded with 160 ng of the libraries three times during a run of 72 h total, with washing steps in between using the Flow Cell Wash Kit EXP-WSH003 (Oxford Nanopore Technologies).

After sequencing, high-accuracy base calling of the raw reads was performed with an optimized version of Guppy v6.0.1 (Oxford Nanopore Technologies) (dna_r9.4.1_450bps_hac.cfg model), resulting in a total of 25 Gb sequence data (~ 190 × genome coverage). The resulting reads were de novo assembled using Flye v2.7.1 [124] with setting minimum overlap as 10 kb and with the “–meta” option, followed by four rounds of polishing with Racon v1.3.3 [125] starting from the Flye assembly with option (-m 8 -x -6 -g -8 -w 500). After each polishing round, reads were re-aligned to the resulting assembly with minimap2 v2.17 [126]. A final round of polishing was performed using Medaka v1.2.0 (https://github.com/nanoporetech/medaka) with the r941_min_high_g344 model using the MinION raw reads. After polishing, haplotype redundancies were merged using Purge haplotigs v1.0.4 [127] and duplicated haplotigs collapsed using Haplomerger2 v2.01 [128].

Illumina short-read genome sequencing was performed at the National Genomics Infrastructure (NGI, Science for Life Laboratory), Stockholm, Sweden. Genomic DNA was extracted from a single male and a single female *T. lineatum*. The DNA samples were used to generate 10X Chromium libraries that were sequenced (paired-end 2 × 150 bp, 1 lane) using a NovaSeq6000 system (Illumina) with a 2 × 151 setup using ‘NovaSeqXp’ workflow in ‘SP’ mode flowcell. The sequencing generated 261.27 and 134.79 million reads for the male and female sample, respectively. Polishing of the final MinION assembly with Illumina short reads was performed using ntHits ver. 0.1.1(https://github.com/bcgsc/nthits) and ntEdit ver. 1.3.2 [129] using default settings. The relative contig coverage, GC content and contig taxonomic classification were scanned after genome assembly using Blob tools and Taxon Analysis [130] to enable the identification and removal of potential microbial symbionts or contaminants. The completeness of the final assembly was assessed using the Benchmarking Universal Single-Copy Orthologs (BUSCOv4.0.6; https://busco.ezlab.org/) tool performed against the Insecta odb10 datasets that includes 1367 reference genes [131].

### Antennal transcriptome sequencing and assembly

For transcriptome sequencing, adult male and female *T. lineatum* were collected during two field seasons (April-June 2021–2022) using pheromone traps as described above. Antennae collected from 135 individuals (males and females in approx. equal sex ratio) were used for RNA extractions, with antennae from the two sexes pooled to obtain sufficient RNA quantity. The antennae were homogenized using Tissue-tearor model 98,370–365 (Bartlesville, OK, USA). Total RNA was then isolated using the RNeasy Minikit (Qiagen, Hilden, Germany). This yielded 0.94 µg of total RNA that was used for transcriptome sequencing. Illumina short-read sequencing was performed at the NGI (Science for Life Laboratory), Sweden. The RNA was DNAse-treated and subjected to library construction using the Illumina TrueSeq stranded mRNA (polyA) kit (Illumina, San Diego, CA, USA). Libraries were sequenced paired-end (2 × 150 bp) on a NovaSeq S6000 platform using 0.25 lanes of an S4-300 (v.1.5) flowcell. The sequencing generated 576.91 million paired-end reads. Low-quality reads and adaptor sequences were removed prior to the de novo assembly with Trinity (v.2.11.0) [132]. Redundant transcripts were removed using CD-HIT-EST (v.4.8.1) [133] with a sequence identity threshold of 0.98. The non-redundant assembly comprised 102,112 predicted ‘genes’ with their respective isoforms together with other non-coding sequences totalling 154,547 ‘transcripts’. The average transcript length was 1,146 bp. The completeness of the assembly was evaluated using BUSCO (v.4.1.4) as described above. This analysis showed high (96.5%) overall completeness of the assembly, with 39.2% of the genes present as single-copy orthologues and 57.3% of the genes duplicated. Only 37 BUSCO genes (2.7%) were missing from the assembly and 11 genes (0.8%) were fragmented. The assembled antennal transcriptome was used to verify the chemoreceptor gene sequences annotated from the genome (described below) and to investigate whether their transcripts are present in the main chemosensory organ, but not for gene annotation per se*.*

### Chemosensory gene annotation

Exhaustive tBLASTn searches were carried out against the polished MinION genome assembly of *T. lineatum* to identify the chemoreceptor genes, using query sequences from the bark beetles *I. typographus* and *D. ponderosae*, as well as the cerambycid *A. glabripennis* [35, 52, 84]. The protein sequences of the identified *T. lineatum* chemoreceptors were then used in additional blast searches against the *T. lineatum* genome until all novel hits were exhausted. The blast searches were implemented in Geneious prime v.11.0.18 software (Biomatters Ltd., Auckland, New Zealand) with an E-value cut-off at 3 (or 10, occasionally for divergent GRs and IRs) to ensure that divergent genes were not missed. Gene annotations and determination of exon/intron boundaries were performed manually in Geneious software as in previous studies [34, 35]. The annotated gene models were further validated through BLAST searches against the non-redundant protein collection at NCBI and against the assembled antennal transcriptome of *T. lineatum* (using tBLASTn), as described above.

The naming of the ORs, most GRs, and the ‘divergent’ IRs followed previously established nomenclature guidelines and was based on two criteria [35]. First, preliminary trees were constructed to assign numbers to genes based on their phylogenetic positions, with related genes given consecutive numbers. Secondly, genes present in tandem arrays on contigs were assigned consecutive numbers, and such genes generally also grouped together in the trees. The conserved carbon dioxide receptors were named GR1-3 (according to [134]), a putative non-fructose sugar receptor was named GR4, and one putative fructose receptor GR5. The numbering of putative bitter taste GRs started at GR6. The numbering of ‘divergent' IRs started at IR101, whereas the conserved ‘antennal’ IRs were named based on orthology with IRs in *Drosophila melanogaster*, except for the IR75 members that were named based on orthology with IR75 proteins in *D. ponderosae* and/or *H. hampei*. Genes that were lacking the N-terminal or C-terminal were designated with the suffixes NTE, and CTE respectively. Because Nanopore sequencing technology is prone to introducing indels in homopolymer regions, some of the chemoreceptor genes contained single nucleotide insertions or deletions inside one or more exons. These indels were manually corrected with support from the Illumina reads, and the corresponding genes attained a FIX suffix. Genes with premature stop codons, frameshifts, missing splice sites and/or missing exons (or pieces of exons) were considered pseudogenes and were given a PSE suffix. Chemoreceptor genes assembled on some of the shortest contigs that shared an amino acid identity > 96% with genes assembled on larger contigs were considered likely assembly variants (or alleles) and were thus not further considered to prevent overestimation of gene counts.

### Phylogenetic analysis

To investigate the evolutionary relationships among the identified chemoreceptors in *T. lineatum* and receptors from other beetles (from Curculionidae and Cerambycidae), we constructed approximately maximum likelihood trees that contained the receptors from *T. lineatum* alongside those previously annotated from the genomes of *D. ponderosae* [35], *H. hampei* [53] and *A. glabripennis* [52]. In the case of ORs, we also included the functionally characterised receptors from *I. typographus* to infer functions in potentially related TlinORs [83, 84, 115, 116]. Multiple sequence alignments were constructed using MAFFT v7.490 [135]. Minor misalignments of a limited number of receptors were manually corrected. The alignments were then trimmed to discard uninformative regions using trimAL v.1.2 [136] with the following settings: similarity threshold 0, gap threshold 0.7, and minimum 25% conserved positions. The trees were constructed using FastTree 2.1.11 (implemented in Geneious Prime software). Local branch support was assessed using the Shimodaira-Hasegawa (SH) test within FastTree. The OR tree was rooted with the conserved Orco lineage, the GR tree with the ancestral clade of sugar receptors, and the IR tree with the conserved IR8a/IR25a lineage. The trees were visualized and colour-coded using FigTree v1.4.4. Final graphical adjustments were made using Adobe Illustrator.

### Supplementary Information

Additional file 1. Amino acid sequences and annotation details of the chemosensory genes identified in the genome of *Trypodendron lineatum*. Information on genomic location, gene structure, presence in antennal transcriptome, and protein length is presented alongside relevant annotation notes.

Additional file 2. The observed simple (1:1) orthologous relationships between chemoreceptors across the analysed species (*Trypodendron lineatum*, *Dendroctonus ponderosae*, *Hypothenemus hampei*, and *Anoplophora glabripennis*).

## Data Availability

The datasets supporting the conclusions of this study are included in this article and its Additional files. The genome sequence data for this study have been deposited in the European Nucleotide Archive (ENA) at EMBL-EBI under the accession number PRJEB74033 (https://www.ebi.ac.uk/ena/browser/view/PRJEB74033) [137]. The antennal RNAseq reads have been deposited in the SRA database at NCBI under the accession number PRJNA1126204 (https://www.ncbi.nlm.nih.gov/sra/PRJNA1126204) [138].
